# High-altitude thrombosis: divergent pathophysiological mechanisms and preventive strategies in acutely exposed population and native highlanders

**DOI:** 10.3389/fcvm.2026.1752475

**Published:** 2026-03-17

**Authors:** Meiquan Li, Jinrui Guo, Meiwei Zhao, Yalan Han, Fuqing Ji, Zichao Liu

**Affiliations:** 1 College of Agriculture and Life Sciences, Kunming University, Kunming, Yunnan, China; 2 Department of Cardiac Arrhythmia, Fuwai Yunnan Cardiovascular Hospital, Kunming Medical University, Kunming, Yunnan, China; 3 College of Food Science and Technology, Yunnan Agricultural University, Kunming, Yunnan, China; 4 Kunming Kingmed Institute for Clinical Laboratory Co., Ltd. Kunming, Kunming, Yunnan, China

**Keywords:** HIF-1*α*, high-altitude, hypoxia, inflammation, thrombosis

## Abstract

**Purpose of review:**

High-altitude environments pose distinct physiological challenges, with marked differences in the thrombotic responses between high-altitude acutely exposed individuals (HAAEI) and long-term high-altitude natives (HAN). This narrative review explores the divergent mechanisms underlying thrombosis in these two populations, aiming to deepen understanding of hypoxia-induced vascular risk.

**Recent findings:**

Acute high-altitude exposure elicits a cascade of responses—including sympathetic overactivation, inflammation, polycythemia, increased blood viscosity, and endothelial dysfunction—that collectively heighten thrombosis risk. Central to this process is the hypoxia-mediated interaction of HIF with NF-*κ*B pathways, which fosters a proinflammatory and procoagulant milieu characterized by cytokine upregulation, enhanced platelet activation, and suppressed fibrinolysis. Conversely, HAN exhibit genetic and physiological adaptations—such as improved oxygen utilization and hematological efficiency—that confer relative protection. However, extreme hypoxia or dehydration can override these adaptations, triggering pathological hyperviscosity. Prevention strategies for HAAEI include gradual acclimatization, hydration, and activity moderation, while HAN may benefit from routine hematological monitoring and, if necessary, antithrombotic interventions.

**Plain language summary:**

Hypoxia-induced alterations in inflammation and blood rheology play pivotal roles in high-altitude thrombosis. Population-specific prevention strategies are essential, and further research is needed to refine pathophysiological understanding and guide targeted interventions.

## Introduction

High altitude, typically considered as areas at or above 2,500 m elevation, presents a set of extreme environmental stressors, including low barometric pressure, intense solar radiation, frigid temperatures, and dry air (1, 2). Together, these factors impose significant physiological challenges, particularly during the initial phase of exposure before adequate acclimatization occurs. People who suddenly travel or work at high elevation, such as tourists, workers, or soldiers, often develop acute altitude-associated disorders, notably acute mountain sickness (AMS), high-altitude cerebral edema (HACE), and high-altitude pulmonary edema (HAPE) (3–5). Epidemiological data indicate that HAPE affects about 4% of Himalayan trekkers and Alpine climbers (6), and HACE occurs in 0.5%–1.0% of individuals at altitudes between 4,000 and 5,000 m (7).

In addition to these well-characterized altitude illnesses, rapid ascent to extreme elevations has been associated with severe thrombotic events such as cerebral thrombosis (8). A notable study reported that Indian soldiers stationed at high altitudes experienced a 30-fold higher risk of spontaneous vascular thrombosis and stroke after extended exposure (9). In addition to environmental hypoxia, traditional risk factors such as smoking, inherited thrombophilia, and oral contraceptive use can synergistically exacerbate thrombotic risk at high altitude. In contrast, high-altitude natives (HAN), who have resided in such environments over generations, display remarkable physiological resilience (10). This resilience is attributed to long-term genetic and phenotypic adaptations shaped by natural selection, enabling these populations to endure hypoxic and cold stress more effectively (11).

As a narrative review, this article integrates molecular, physiological, and epidemiological perspectives to compare thrombotic mechanisms and preventive strategies in acutely exposed individuals and native highlanders. It aims to elucidate the thrombotic mechanisms occurring in both transient and permanent high-altitude populations. By integrating insights from molecular biology, hemodynamics, and evolutionary physiology, we differentiate between adaptive and maladaptive responses to environmental hypoxia and highlight population-specific risk factors and preventive strategies.

### Physiological mechanisms of thrombosis risk in high-altitude acutely exposed individuals (HAAEI)

Short-term residents at high altitude, such as tourists or temporary workers, face an increased risk of thrombosis due to insufficient physiological adaptation to hypoxic conditions. At high altitudes, the drop in atmospheric pressure reduces the oxygen partial pressure along the entire transport pathway from the surrounding air to its final use in mitochondria (12, 13). Although hypobaric hypoxia typically triggers compensatory responses, such as hyperventilation and erythropoietin (EPO) upregulation, to enhance oxygen delivery (14), maladaptive outcomes often occur, including AMS, thrombosis, HAPE, and HACE (15–17). Research indicates that people from low-altitude regions who travel to high elevations have a markedly increased risk of developing venous thromboembolism (VTE) (18). A five-year retrospective study of U.S. military academies showed that thromboembolic events occurred at twice the rate at 2,210 m elevation compared to sea level (19). Symptoms may arise within hours to days post-ascent and can rapidly escalate. The interplay between hypoxic stress and systemic inflammation disrupts the balance between coagulation and fibrinolysis, exacerbating thrombotic risk (20). Acutely exposed individuals experience a transient yet critical “thrombotic window” during early high-altitude exposure, necessitating proactive surveillance and intervention.

### Sympathetic activation and hemoconcentration

Acute hypoxia triggers the sympathetic nervous system to release epinephrine and norepinephrine from the adrenal medulla. This leads to a faster heart rate, stronger heart contractions, and narrowing of peripheral blood vessels to ensure blood flow to essential organs like the brain and heart (21–23). Simultaneously, hypoxia-induced hyperventilation increases respiratory water loss, causing a substantial reduction in plasma volume (8%–15% within 48 h) and hemoconcentration (hematocrit increase of 5%–10%), thereby raising blood viscosity by 20%–30% (24–26). This hyperviscous state, coupled with impaired venous return and heightened erythrocyte and platelet aggregation, creates a critical prothrombotic phase peaking between 48 and 72 h post-exposure.

### Erythrocytosis

In response to hypoxia, EPO levels rise sharply within hours of exposure and peak around 48 h, sustaining above baseline for several weeks (27). This stimulates erythroid progenitor differentiation in the bone marrow, leading to increased circulating reticulocytes detectable within 72 h (28). While reticulocyte-to-erythrocyte maturation typically completes within 96 h at sea level, high-altitude conditions induce two distinct perturbations: (1) premature release of reticulocytes due to increased bone marrow output pressure (29); and (2) prolonged maturation duration (> 120 h) attributed to oxidative stress-mediated impairment in erythroblast enucleation (30). The resulting erythrocytosis manifests differently in acutely exposed individuals and native highlanders. In acclimatizing lowlanders, it is primarily a reactive, EPO-driven process aimed at rapidly enhancing oxygen carriage, often leading to a significant but transient rise in hematocrit (31). In contrast, native highlanders, particularly those with adaptive genetic backgrounds, exhibit a more stabilized erythrocytosis. This is influenced by protective haplotypes in HIF-pathway genes, such as EPAS1 and EGLN1, which modulate the erythropoietic response and are associated with a blunted or optimized hemoglobin phenotype, preventing the detrimental effects of excessive erythrocytosis (32). These erythropoietic adaptations function concurrently with broader physiological adjustments to maintain oxygen homeostasis (24, 33). Since maintaining oxygen balance is vital for survival, sudden exposure to high-altitude hypoxia causes plasma volume to decrease, while total red blood cell volume and hemoglobin mass gradually rise (34). This response is crucial, as it stimulates erythropoietic drive. These physiological adaptations collectively preserve overall blood volume while increasing hemoglobin concentration, thereby enhancing oxygen-carrying capacity (35, 36). Researchers have found that spending two weeks at moderate altitude does not significantly raise hemoglobin levels in athletes (37), whereas prolonged exposure of over three weeks results in a steady increase in total red blood cell volume, ranging from 60 to 250 mL per week (38). The cumulative impact of these changes—elevated blood viscosity and reduced flow velocity—alongside endothelial dysfunction and inflammatory activation, intricately elevates the risk of thrombosis under chronic hypoxic stress. Notably, accumulating evidence suggests that EPO itself may act as a direct risk factor for thrombotic events, independent of its indirect effects via erythrocytosis (39, 40).

### Inflammatory response and imbalance of coagulation-fibrinolysis system

High-altitude thrombosis is mediated by a complex interplay between hypoxia, inflammation, and coagulation, largely coordinated through hypoxia-inducible factor 1-alpha (HIF-1*α*) (41–45). HIF-1*α* serves as a critical transcription factor, orchestrating cellular adaptation to low oxygen levels by modulating gene expression (46, 47). Under hypoxic conditions, inhibition of prolyl hydroxylase (PHD) stabilizes HIF-1*α*, allowing it to activate gene transcription for cellular adaptation (48). Acute exposure not only induces neutrophilia and elevates pro-inflammatory cytokines and neutrophil chemokines (49, 50), but also promotes neutrophil survival via HIF-1*α*-dependent NF-*κ*B activation (51). HIF-1*α* and NF-*κ*B interact in a reciprocal manner; HIF regulates NF-*κ*B transcription (52); conversely, NF-*κ*B induces HIF-1*α* expression by enhancing HIF-1*α* mRNA stability (53). Under normoxic conditions, NF-*κ*B activity is suppressed by its interaction with the inhibitory protein I-*κ*B. Hypoxic conditions lead to I-*κ*B kinase (IKK) phosphorylation of I-*κ*B, triggering its degradation and NF-*κ*B nuclear translocation (54). This reciprocal regulation is exemplified by studies showing that IKK-*β* knockdown impedes HIF-1*α* transcriptional activity, and hypoxia-mediated IKK-*β* suppression paradoxically boosts NF-*κ*B signaling (55). Furthermore, NF-*κ*B and HIF-1*α* synergistically escalate inflammatory responses, as hypoxia enhances TAK1/NF-*κ*B/HIF-1*α* signaling, significantly increasing IL-6 and IL-8 production by 3-5-fold (56). A study found that dabigatran etexilate reduces the hypercoagulable state by regulating coagulation, improving endothelial function of rats, and influencing the NF-*κ*B/IL-1β/MCP-1 and TLR4/NLRP3 signaling pathways (57). Studies have shown that suppressing oxidative stress and the TLR4/NF-*κ*B/NLRP3 signaling pathway can effectively reduce acute lung injury in rats subjected to high-altitude hypoxia (58). Hypobaric hypoxia significantly elevates the levels of inflammatory cytokines, such as TNF-α, IL-1β, and IL-6, as well as corticotropin-releasing hormone in both human and animal plasma (59).

**Figure 1:**
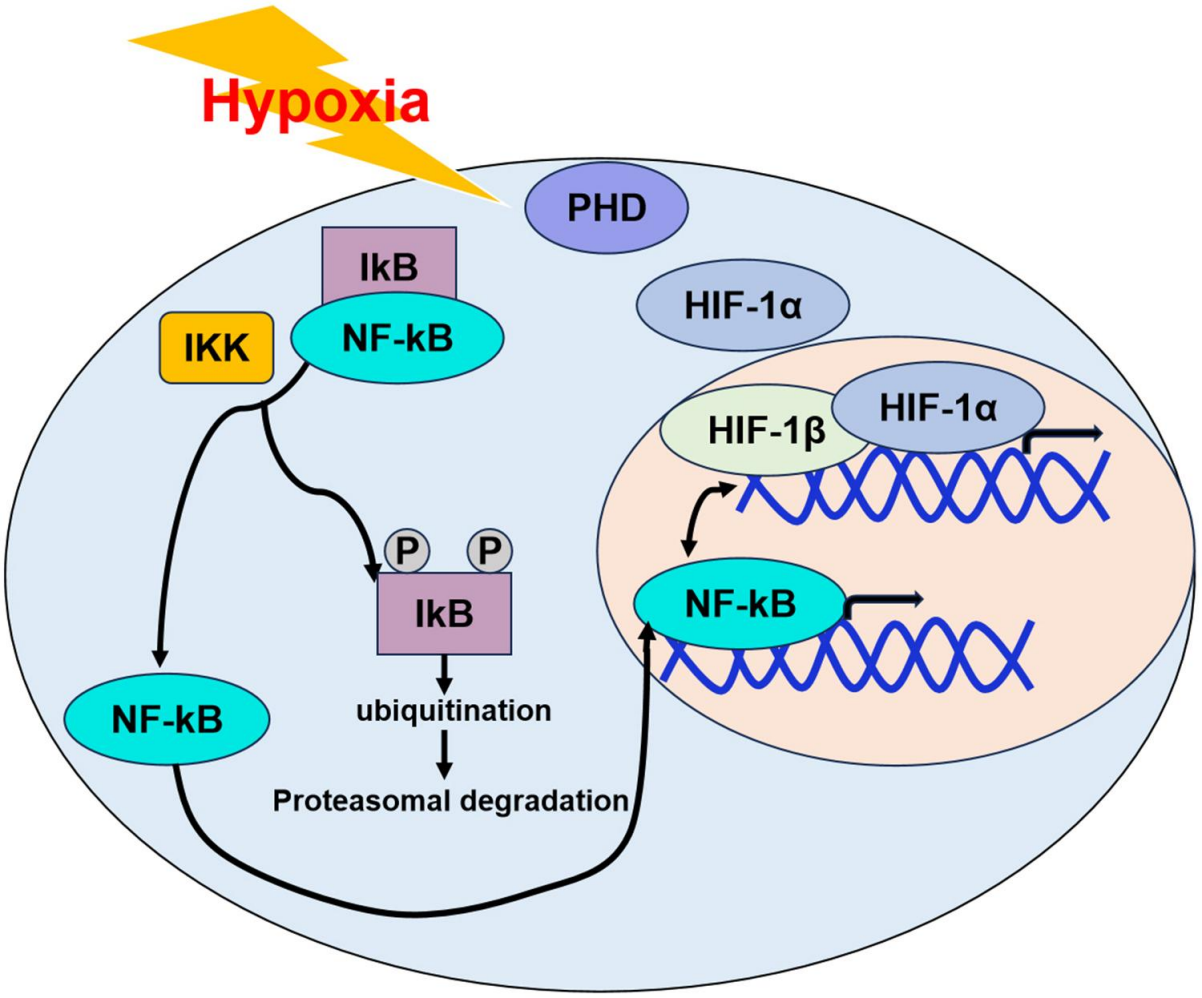
Synergistic activation of inflammatory signaling by HIF-1*α* and NF-*κ*B under acute high-altitude hypoxia.

The increases are positively associated with both the occurrence and severity of hypercoagulation. Simultaneously, these interdependent pathways establish a feedforward loop, perpetuating inflammation under hypoxic conditions. This intricate interplay likely drives systemic inflammation in acute high-altitude hypoxemia through both direct cytokine release and epigenetic reprogramming of immune cells. Acute hypobaric hypoxia at high altitudes inhibits prolyl hydroxylase (PHD) activity, triggering two interconnected pathways: (1) Stabilization of HIF-1*α* due to impaired oxygen-dependent degradation, which activates hypoxia adaptation mechanisms; (2) Augmentation of I*κ*B kinase (IKK) activity resulting from decreased hydroxylation, facilitating phosphorylation and proteasomal degradation of I*κ*B. This dual mechanism releases NF-*κ*B dimers (p50/p65) for nuclear translocation, where they synergize with HIF-1*α* to amplify transcription of proinflammatory mediators (IL-6, IL-8, TNF-α) (Figure 1).

Inflammation induces coagulation activation through multiple interconnected pathways. These pathways generally include cytokine-driven Tissue Factor (TF) expression, inflammation-related reduction of the protein C system, and inhibition of fibrinolysis. Specifically, cytokine IL-6 directly promotes TF production, and experimental studies in animal models of septic shock demonstrate that inhibiting either TF or IL-6 effectively prevents thrombin elevation (60, 61). Besides IL-6, pro-inflammatory cytokines like TNF-α and IL-1β have also been found to stimulate TF expression (62, 63). High-altitude hypoxia induces a prothrombotic propensity via the activation of calpain and the enhancement of the inflammatory complex, thereby increasing reactive thrombosis in response to hypoxia (41, 64). Moreover, the inflammation-driven upregulation of TF synthesis is not confined to immune cells; endothelial cells, monocytes/macrophages, and dendritic cells also exhibit heightened TF production under inflammatory conditions (65, 66). Simultaneously, a self-perpetuating pathological loop emerges from interactions between platelets, leukocytes, and endothelial cells that both amplify systemic inflammation and increase procoagulant activity, creating a self-reinforcing pathological loop (67, 68). The fibrinolytic system is central to the link between inflammation and coagulation, serving under normal conditions to preserve balance. Under normal conditions, plasminogen activator inhibitor-1 (PAI-1) regulates fibrinolysis by blocking major activators such as tissue-type plasminogen activator (tPA) and urokinase-type plasminogen activator (uPA) (69). However, inflammatory stress provokes overproduction of PAI-1 in endothelial cells, disrupting the fibrinolytic balance and promoting thrombus formation by perpetuating fibrin deposition (70). PAI-1 polymorphism has been identified as a factor contributing to severe arteriovenous thrombosis associated with high altitude (71). This highlights the complex interplay between inflammatory mediators and clotting factors in thrombogenesis.

### Vascular endothelial dysfunction

During brief high-altitude excursions, the abrupt onset of acute hypoxia triggers endothelial dysfunction, primarily through the suppression of nitric oxide (NO) synthesis in endothelial cells (47, 72), thereby promoting oxidative stress and eliciting the release of proinflammatory cytokines, notably IL-6 and TNF-α (73). These pathophysiological changes manifest as diminished vasodilatory capacity, disruption of the endothelial barrier, and a shift towards a procoagulant state, notably marked by elevated TF expression and enhanced platelet activation (74). Furthermore, endothelial damage exposes subendothelial collagen, directly triggering the coagulation cascade, while compromised fibrinolysis, exemplified by increased (PAI-1) levels, further exacerbates this imbalance (75, 76). Collectively, these mechanisms amplify the thrombotic risk within a narrow timeframe.

### Physiological mechanisms underlying thrombotic risk in long-term high-altitude natives (HAN)

Worldwide, approximately 140 million people inhabit regions situated above 2,500 m in elevation (76). Notably, individuals who have adapted to high-altitude environments over extended periods, such as long-term plateau residents, demonstrate a reduced thrombotic risk compared to those acutely exposed to such conditions (77). This reduced risk is attributed to physiological adaptations to sustained high-altitude living, including optimized oxygen utilization and vascular regulation (78). An ecological study in Switzerland found that stroke mortality decreases with higher altitude, showing a 12% reduction for every 1,000 m increase in elevation. The study assessed the effects of altitude on cardiovascular and cerebrovascular diseases using sociodemographic information, birthplaces, and residences of men and women aged 40–84 living between 259 and 1960 m above sea level. The protective effect of living at higher altitudes on coronary heart disease and stroke mortality remained consistent and became stronger after controlling for potential confounding factors (79). Hematological adaptations are also evident in populations with prolonged exposure to varying altitudes. Indigenous Tibetans residing at lower altitudes for more than 50 years show notably reduced red blood cell counts, hematocrit, hemoglobin levels, mean corpuscular hemoglobin, and mean corpuscular hemoglobin concentration, along with an increase in mean corpuscular volume, indicating physiological adaptations to the higher oxygen levels found at lower elevations (80). However, the benefits of high-altitude adaptation are context-dependent, and residing at altitudes exceeding 3,500 m has been associated with a markedly elevated risk of stroke (81, 82). Previous studies have shown that spending one year at high altitude increases the risk of thromboembolic events, such as Deep Vein Thrombosis (DVT) and pulmonary embolism, by 30 times (83). Compared to low-altitude areas, long-term exposure to high altitudes is linked to a higher risk of stroke and related hospitalizations, with rates of 1.05 per 1,000 vs. 13.7 per 1,000 people, respectively (84). This unexpected risk does not stem from erythrocytosis alone, while erythrocytosis increases blood viscosity, it acts in synergy with other factors (such as endothelial dysfunction and inflammation) to precipitate thrombosis. For instance, in high-altitude Andean populations, only 4.5% of patients with erythrocytosis and normal erythropoietin levels develop thrombotic events. Instead, it arises from the progression of excessive erythrocytosis to a maladaptive state, coupled with increased blood viscosity and vascular strain, which together raise the likelihood of thrombosis (80, 85).

### The viscosity of the blood increases

Prolonged residency at high altitudes can induce severe hypoxemia, which is partially mitigated by respiratory adaptations, including those occurring during sleep. However, severe hypoxemia stimulates red blood cell production, causing a significant rise in total hemoglobin concentration and a simultaneous decrease in plasma volume. This combination enhances blood viscosity (86, 87). Crucially, blood viscosity is strongly influenced by hematocrit and hemoglobin concentrations, which progressively increase with altitude. As a result, blood viscosity tends to rise in proportion to altitude. For instance, a recent study in La Rinconada (5,100 m) found that healthy high-altitude residents exhibited red blood cell volumes and hemoglobin mass approximately double those of sea-level inhabitants, a direct adaptation to chronic hypoxia (88). Further research found that residents of La Rinconada have significantly higher blood viscosity than those living in Puno (3,800 m), with both high-altitude groups exhibiting increased viscosity compared to lowland populations (89). Additionally, blood viscosity is influenced by the flow characteristics of red blood cells, such as their ability to aggregate and deform, along with the viscosity of the plasma (90). The clinical ramifications of these changes are profound. Elevated blood viscosity reduces blood flow velocity, particularly in venous circulation, where sluggish flow heightens the likelihood of blood component precipitation and aggregation, key steps in thrombus formation. For example, in pathological conditions like polycythemia vera, excessive red blood cell concentrations elevate viscosity (91). When combined with concurrent vascular endothelial damage, this creates a “perfect storm” that may precipitate arterial thrombosis and, in severe cases, myocardial infarction. In summary, for native high-altitude populations, the relationship between erythrocytosis and thrombotic risk is highly heterogeneous. It is important to distinguish between adapted inhabitants with physiological erythrocytosis and non-adapted individuals with maladaptive phenotypes. For instance, only an estimated 4.5% of Andeans residing at 4,000 m develop excessive erythrocytosis associated with Chronic Mountain Sickness (CMS); this non-adapted subgroup faces significantly elevated blood viscosity and a higher risk of developing thrombosis (85). Within this susceptible subgroup, the synergy of a markedly increased red cell mass, microcirculatory dysfunction, platelet activation, endothelial injury, and chronic inflammation can promote a hypercoagulable state, thereby amplifying the risk of both arterial and venous thrombosis.

### Variations in individual adaptability

Differences in individual adaptability to high-altitude environments are markedly apparent among plateau-native populations. For instance, Tibetans have evolved unique genetic adaptations, such as variants in the EPAS1 and EGLN1 genes, enabling them to maintain lower hemoglobin concentrations compared to other high-altitude populations (92–94). This adaptation not only reduces blood viscosity but also minimizes the risk of thrombosis, thereby conferring a survival advantage in hypoxic conditions. Beyond genetic factors, prolonged exposure to high-altitude environments may further shape physiological resilience through epigenetic mechanisms. Specifically, emerging research in humans and snub-nosed monkeys suggests that chronic hypoxia can influence the expression of coagulation-related genes through processes such as DNA methylation (95, 96). These epigenetic modifications may fine-tune hemostatic balance, thereby potentially enhancing adaptability to sustained low-oxygen stress.

### Strategies for the prevention and treatment of thrombosis at high altitudes

The primary health risks associated with high-altitude exposure stem from chronic hypoxia-induced oxidative stress, inflammatory responses, hemorheological alterations, and coagulation-fibrinolysis imbalances. These challenges call for multi-target synergistic interventions for effective prevention. Initial steps include staged acclimatization (daily ascent of ≤500 m) that systematically activates the HIF pathway to prevent excessive erythrocytosis. Pharmacological preconditioning with agents such as acetazolamide enhances ventilatory efficiency and reduces the risk of cerebral edema. After high-altitude exposure, diastolic, systolic, and mean blood pressures increased significantly in placebo-treated subjects but not in those receiving acetazolamide, indicating that acetazolamide may interfere with sympathetic vasoconstriction and promote vasodilation (97). Moreover, antioxidants, including N-acetylcysteine, along with anti-inflammatory, omega-3- and polyphenol-rich diets, mitigate oxidative stress and NF-*κ*B–mediated inflammation, preserving vascular endothelial homeostasis (98, 99). Concurrently, maintaining adequate hydration (≥4 L of electrolyte-enriched fluids daily) and administering low-dose antiplatelet therapy (e.g., aspirin) modulate blood viscosity and coagulation propensity (100). NO pathway enhancers (e.g., sildenafil) improve microcirculatory perfusion (101, 102). An animal study shows that sildenafil reduces pulmonary hypertension by suppressing inflammatory cytokine expression and activating the protective Akt signaling pathway (103). The arginine/NO pathway modulates both erythropoiesis and vasodilation. Studies have demonstrated that deletion of mitochondrial arginase 2 enhances cardiovascular function and mitigates hypoxia-induced pulmonary hypertension in HANs (104). Furthermore, for genetically predisposed individuals, genetic screening informs personalized monitoring strategies. Emerging epigenetic interventions may reverse hypoxia-driven hypercoagulable phenotypes. Experiments in mice have shown that targeting the transferrin-coagulation pathway has been identified as a promising and effective approach to prevent thromboembolic events triggered by adverse environmental factors at high altitude (40, 105).

However, human studies suggest that elevated transferrin may instead confer protection against thrombosis (106, 107). This protective effect of elevated transferrin against thrombosis has also been recently observed in sickle cell disease, another condition characterized by heightened hypoxic responses due to anemia and microvascular occlusion (108). This contrast is particularly noteworthy, as the hypoxia in sickle cell disease originates from hematological disorders rather than environmental exposure, further highlighting that the role of transferrin may vary depending on the underlying cause of the hypoxic state. The observed differences may be attributed to physiological variations underlying divergent hypoxia responses and altitude adaptations among humans, rats, and mice (109). Behaviorally, nocturnal intermittent oxygen supplementation and avoidance of alcohol or strenuous activity diminish cumulative risks. Future studies should focus on developing HIF-targeted precision therapeutics and integrating gene-environment interaction data to optimize prevention strategies, thereby maintaining a balanced equilibrium between hypoxic adaptation and pathological compensation.

## Conclusion

Thrombosis at high altitude is a multifactorial condition driven by complex alterations in blood composition, vascular function, and the coagulation-fibrinolysis balance. Both HAAEI and HAN are at risk, but the mechanisms are distinct and population-specific (Table 1).

**Table 1:** Differences in thrombosis risk between HAAEI and HAN at high altitude.

Comparison dimension	HAAEI	HAN
Core mechanisms	Acute hypoxia-induced sympathetic overactivation, inflammation-coagulation imbalance, endothelial dysfunction	Chronic hypoxia-driven erythrocytosis, elevated blood viscosity, microcirculatory stasis
Critical molecules/indicators	- HIF-1*α*/NF-*κ*B pathway activation - IL-6, TNF-α↑ - Tissue factor (TF) ↑ - PAI-1 (impaired fibrinolysis) ↑ - NO↓ - Erythropoietin (EPO) ↑	- Erythropoietin (EPO): normal (in adapted individuals); (only in secondary erythrocytosis) ↑ - Hemoglobin: ∼15 g/dL (Tibetans), ∼16.5 g/dL (Andeans); >20 g/dL (only in CMS patients) - Blood viscosity ↑ (30–50% higher than sea-level residents)
Thrombosis types	Predominantly venous (DVT, PE)	Predominantly arterial (MI, stroke), especially with coexisting atherosclerosis
Adaptive differences	Lack of compensatory adaptation; transient “prothrombotic window”	Genetic mutations in natives maintain lower hemoglobin, reducing thrombosis risk
Molecular regulation	- Synergistic activation of inflammatory/coagulation pathways - Impaired endothelial NO synthesis	- DNA methylation-mediated regulation of coagulation factors - Altered microcirculatory hemodynamics (stasis)

↑ indicates elevated levels or upregulation; ↓ indicates reduced levels or downregulation.

In HAAEI, thrombosis is primarily triggered by a transient prothrombotic window, characterized by sympathetic overactivation, hypoxia-induced inflammation, increased blood viscosity, endothelial dysfunction, and activation of HIF-1*α*/NF-*κ*B pathways. In HAN, the risk arises from long-term physiological adaptations, notably erythrocytosis, elevated hemoglobin, and increased blood viscosity, that optimize oxygen delivery but may predispose to thrombotic events under additional stressors such as dehydration or extreme hypoxia. Thus, thrombosis at high altitude represents two distinct pathophysiological models: acute maladaptive responses in HAAEI and chronic compensatory risks in HAN.

Effective prevention requires tailored strategies for each group. For HAAEI, gradual acclimatization, hydration, and pharmacological interventions (e.g., acetazolamide, antioxidants, antiplatelet agents) are crucial to minimize early exposure risks. For HAN, regular hematological monitoring, blood viscosity management, and genetic/epigenetic screening may help prevent long-term thrombotic complications. Future research should prioritize: (1) elucidating population-specific molecular mechanisms (e.g., HIF-pathway regulation, epigenetic modification); (2) identifying biomarkers for thrombotic susceptibility; and (3) developing precision interventions that balance hypoxic adaptation with thrombosis prevention.

By recognizing the divergent mechanisms and implementing population-adapted preventive strategies, we can more effectively mitigate the thrombotic risks associated with high-altitude exposure.

## Methods and evidence synthesis

This narrative review integrates current molecular, physiological, and epidemiological evidence on thrombosis at high altitude. To systematically present the evidence base, we incorporated laboratory studies, clinical observations, epidemiological investigations, and ecological studies. Evidence was selected based on its relevance in elucidating the divergent thrombotic mechanisms between acutely exposed individuals and high-altitude natives (Table 2).

**Table 2:** Summary of evidence types underpinning this review.

Evidence category	Study design/model	Key findings/contributions
Laboratory studies (animal models)	Rat/mouse models of hypoxia, gene knockout models	Elucidated the central role of HIF-1α/NF-κB signaling, inflammasome activation, and oxidative stress in thrombosis; validated mechanisms of potential pharmacological interventions (e.g., dabigatran, sildenafil) (37, 53, 54, 96).
Human physiological studies	Cross-sectional studies, cohort studies, and interventional trials (e.g., drug interventions) in individuals with acute or chronic high-altitude exposure.	Quantified hemodynamic and hemorheological changes (blood viscosity, hematocrit) and inflammatory markers in acutely exposed individuals; evaluated the preventive effects of acclimatization and medications (e.g., acetazolamide) (19, 24, 26, 92).
Epidemiological studies	Retrospective cohort studies, ecological studies, population-based cross-sectional surveys.	Revealed associations between high-altitude exposure and the incidence/mortality of venous thromboembolism (VTE) and stroke; compared disease risks among populations at different altitudes (8, 9, 75, 77, 80).
Genetic & evolutionary biology studies	Genome-wide association studies (GWAS), comparative genomics, epigenetic analyses.	Identified genetic adaptations unique to high-altitude native populations [e.g., Tibetans, EPAS1/EGLN1 variants (32); Andeans, NFKB1, NOS2 (110, 111); Ethiopians, ARNT2, THR B^112^, ^113^] and epigenetic mechanisms potentially fine-tuning coagulation (32, 87–90).
Studies on high-altitude natives	Observational studies of long-term residents of high-altitude regions (e.g., Andes, Tibetan Plateau).	Described unique physiological adaptations in native populations, such as optimized oxygen utilization, relatively lower hemoglobin levels (in Tibetans), or pathological erythrocytosis and its associated risks (10, 76, 83, 84).

## References

[1] Li Y Zhang Y Zhang Y. Research advances in pathogenesis and prophylactic measures of acute high altitude illness. Respir Med. (2018) 145:145–52. 10.1016/j.rmed.2018.11.004 30509704

[2] Basnyat B Murdoch DR. High-altitude illness. Lancet. (2003) 361:1967–74. 10.1016/S0140-6736(03)13591-X 12801752

[3] Richalet JP Hermand E Lhuissier FJ. Cardiovascular physiology and pathophysiology at high altitude. Nat Rev Cardiol. (2024) 21:75–88. 10.1038/s41569-023-00924-9 37783743

[4] Sydykov A Mamazhakypov A Maripov A Kosanovic D Weissmann N Ghofrani HA Pulmonary hypertension in acute and chronic high altitude maladaptation disorders. Int J Environ Res Public Health. (2021) 18(4):1692. 10.3390/ijerph18041692 33578749 PMC7916528

[5] Gallagher SA Hackett PH. High-altitude illness. Emerg Med Clin North Am. (2004) 22:329–55. 10.1016/j.emc.2004.02.001 15163571

[6] Shrestha P Pun M Basnyat B. High altitude pulmonary edema (HAPE) in a Himalayan trekker: a case report. Extrem Physiol Med. (2014) 3:1–4. 10.1186/2046-7648-3-6 24636661 PMC3984695

[7] Bärtsch P Swenson ER. Clinical practice: acute high-altitude illnesses. N Engl J Med. (2013) 368:2294–302. 10.1056/NEJMcp1214870 23758234

[8] Kotwal J Apte CV Kotwal A Mukherjee B Jayaram J. High altitude: a hypercoagulable state: results of a prospective cohort study. Thromb Res. (2007) 120:391–7. 10.1016/j.thromres.2006.09.013 17084442

[9] Anand AC Saha A Seth AK Chopra GS Nair V Sharma V. Symptomatic portal system thrombosis in soldiers due to extended stay at extreme altitude. J Gastroenterol Hepatol. (2005) 20:777–83. 10.1111/j.1440-1746.2005.03723.x 15853994

[10] Beall CM. Two routes to functional adaptation: Tibetan and Andean high-altitude natives. Proc Natl Acad Sci USA. (2007) 104:8655–60. 10.1073/pnas.0701985104 17494744 PMC1876443

[11] Beall CM. Human adaptability studies at high altitude: research designs and major concepts during fifty years of discovery. Am J Hum Biol. (2013) 25:141–7. 10.1002/ajhb.22355 23349118

[12] Smith ZM Krizay E Guo J Shin DD Scadeng M Dubowitz DJ. Sustained high-altitude hypoxia increases cerebral oxygen metabolism. J Appl Physiol (1985). (2013) 114:11–8. 10.1152/japplphysiol.00703.2012 23019310 PMC3544513

[13] Furian M Tannheimer M Burtscher M. Effects of acute exposure and acclimatization to high-altitude on oxygen saturation and related cardiorespiratory fitness in health and disease. J Clin Med. (2022) 11(22):6699. 10.3390/jcm11226699 36431176 PMC9697047

[14] Schmidt W. Effects of intermittent exposure to high altitude on blood volume and erythropoietic activity. High Alt Med Biol. (2002) 3(2):167–76. 10.1089/15270290260131902 12162861

[15] Khodaee M Grothe HL Seyfert JH VanBaak K. Athletes at high altitude. Sports Health. (2016) 8(2):126–32. 10.1177/1941738116630948 26863894 PMC4789936

[16] Gatterer H Villafuerte FC Ulrich S Bhandari SS Keyes LE Burtscher M. Altitude illnesses. Nat Rev Dis Primers. (2024) 10(1):43. 10.1038/s41572-024-00526-w 38902312

[17] Yin X Li Y Ma Y Xie Y Wang K Sun D Thickened retinal nerve fiber layers associated with high-altitude headache. Front Physiol. (2022) 13:864222. 10.3389/fphys.2022.864222 35600299 PMC9114875

[18] Shishir K. High altitude induced deep venous thrombosis: a study of 28 cases. Indian J Surg. (2006) 68(2):84–8. http://hdl.handle.net/1807/6394

[19] Smallman DP McBratney CM Olsen CH Slogic KM Henderson CJ. Quantification of the 5-year incidence of thromboembolic events in U.S. Air force academy cadets in comparison to the U.S. Naval and Military Academies. Mil Med. (2011) 176:209–13. 10.7205/milmed-d-10-00144 21366086

[20] Göbel K Eichler S Wiendl H Chavakis T Kleinschnitz C Meuth SG. The coagulation factors fibrinogen, thrombin, and factor XII in inflammatory disorders-A systematic review. Front Immunol. (2018) 9:1731. 10.3389/fimmu.2018.01731 30105021 PMC6077258

[21] Bärtsch P Gibbs JS. Effect of altitude on the heart and the lungs. Circulation. (2007) 116(19):2191–202. 10.1161/circulationaha.106.650796 17984389

[22] Hainsworth R Drinkhill MJ Rivera-Chira M. The autonomic nervous system at high altitude. Clin Auton Res. (2007) 17:13–9. 10.1007/s10286-006-0395-7 17264976 PMC1797062

[23] Sander M. Does the sympathetic nervous system adapt to chronic altitude exposure? Hypoxia. (2016) 903:375–93. 10.1007/978-1-4899-7678-9_25 27343109

[24] Simonson TS. Altitude adaptation: a glimpse through various lenses. High Alt Med Biol. (2015) 16(2):125–37. 10.1089/ham.2015.0033 26070057 PMC4490743

[25] Weil JV Byrne-Quinn E Sodal IE Friesen WO Underhill B Filley GF Hypoxic ventilatory drive in normal man. J Clin Invest. (1970) 49(6):1061–72. 10.1172/jci106322 5422012 PMC322574

[26] Heinicke K Prommer N Cajigal J Viola T Behn C Schmidt W. Long-term exposure to intermittent hypoxia results in increased hemoglobin mass, reduced plasma volume, and elevated erythropoietin plasma levels in man. Eur J Appl Physiol. (2003) 88(6):535–43. 10.1007/s00421-002-0732-z 12560952

[27] Berglund B Gennser M Ornhagen H Ostberg C Wide L. Erythropoietin concentrations during 10 days of normobaric hypoxia under controlled environmental circumstances. Acta Physiol Scand. (2002) 174(3):225–9. 10.1046/j.1365-201x.2002.00940.x 11906321

[28] Brugnara C. Reticulocyte cellular indices: a new approach in the diagnosis of anemias and monitoring of erythropoietic function. Crit Rev Clin Lab Sci. (2000) 37(2):93–130. 10.1080/10408360091174196 10811141

[29] Edwards MJ Novy MJ Walters C-L Metcalfe J. Improved oxygen release: an adaptation of mature red cells to hypoxia. J Clin Invest. (1968) 47(8):1851–7. 10.1172/JCI105875 5666114 PMC297345

[30] Tang S Zhou W Chen L Yan H Chen L Luo F. High altitude polycythemia and its maladaptive mechanisms: an updated review. Front Med (Lausanne). (2024) 11:1448654. 10.3389/fmed.2024.1448654 39257892 PMC11383785

[31] Mairbäurl H Gassmann M Muckenthaler MU. Geographical ancestry affects normal hemoglobin values in high-altitude residents. J Appl Physiol (1985). (2020) 129:1451–9. 10.1152/japplphysiol.00025.2020 33002380

[32] Zhao Y Zhang Z Liu L Zhang Y Fan X Ma L Associations of high altitude polycythemia with polymorphisms in EPAS1, ITGA6 and ERBB4 in Chinese Han and Tibetan populations. Oncotarget. (2017) 8:86736–46. 10.18632/oncotarget.21420 29156832 PMC5689722

[33] Ding XH Wang Y Cui B Qin J Zhang JH Rao RS Acute mountain sickness is associated with a high ratio of endogenous testosterone to estradiol after high-altitude exposure at 3,700 m in young Chinese men. Front Physiol. (2018) 9:1949. 10.3389/fphys.2018.01949 30740062 PMC6355701

[34] Siebenmann C Robach P Lundby C. Regulation of blood volume in lowlanders exposed to high altitude. J Appl Physiol. (2017) 123(4):957–66. 10.1152/japplphysiol.00118.2017 28572493

[35] Feng S Wei G Yang X Zhang Z Qu J Wang D Changes in expression levels of erythrocyte and immune-related genes are associated with high altitude polycythemia. BMC Med Genomics. (2023) 16(1):174. 10.1186/s12920-023-01613-9 37507679 PMC10375625

[36] Mairbäurl H. Red blood cell function in hypoxia at altitude and exercise. Int J Sports Med. (1994) 15(2):51–63. 10.1055/s-2007-1021020 8157369

[37] Friedmann B Jost J Rating T Weller E Werle E Eckardt KU Effects of iron supplementation on total body hemoglobin during endurance training at moderate altitude. Int J Sports Med. (1999) 20(2):78–85. 10.1055/s-2007-971097 10190766

[38] Sawka MN Convertino VA Eichner ER Schnieder SM Young AJ. Blood volume: importance and adaptations to exercise training, environmental stresses, and trauma/sickness. Med Sci Sports Exerc. (2000) 32(2):332–48. 10.1097/00005768-200002000-00012 10694114

[39] Gupta N Ashraf MZ. Exposure to high altitude: a risk factor for venous thromboembolism? Paper/poster presented at: seminars in thrombosis and hemostasis. Semin Thromb Hemost. (2012) 38(2):156–63. 10.1055/s-0032-1301413 22422330

[40] Amaru R Mamani LF Mancilla E Paton D Valencia JC Amaru A Transferrin and erythropoietin increased levels correlate with thrombosis at high altitude. Blood. (2023) 142:5548. 10.1182/blood-2023-188302

[41] Gupta N Sahu A Prabhakar A Chatterjee T Tyagi T Kumari B Activation of NLRP3 inflammasome complex potentiates venous thrombosis in response to hypoxia. Proc Natl Acad Sci U S A. (2017) 114(18):4763–8. 10.1073/pnas.1620458114 28420787 PMC5422823

[42] Prabhakar A Chatterjee T Bajaj N Tyagi T Sahu A Gupta N Venous thrombosis at altitude presents with distinct biochemical profiles: a comparative study from the Himalayas to the plains. Blood Adv. (2019) 3(22):3713–23. 10.1182/bloodadvances.2018024554 31765479 PMC6880906

[43] Harris AJ Thompson AR Whyte MK Walmsley SR. HIF-mediated innate immune responses: cell signaling and therapeutic implications. Hypoxia (Auckl). (2014) 2:47–58. 10.2147/hp.S50269 27774466 PMC5045056

[44] Gangwar A Pooja, Sharma M Singh K Patyal A Bhaumik G Bhargava K Intermittent normobaric hypoxia facilitates high altitude acclimatization by curtailing hypoxia-induced inflammation and dyslipidemia. Pflugers Arch. (2019) 471(7):949–59. 10.1007/s00424-019-02273-4 30980137

[45] Pham K Vargas A Frost S Shah S Heinrich EC. Changes in immune cell populations during acclimatization to high altitude. Physiol Rep. (2024) 12(22):e70024. 10.14814/phy2.70024 39551933 PMC11570420

[46] Semenza GL. Regulation of oxygen homeostasis by hypoxia-inducible factor 1. Physiology (Bethesda). (2009) 24:97–106. 10.1152/physiol.00045.2008 19364912

[47] Fan N Liu C Ren M. Effect of different high altitudes on vascular endothelial function in healthy people. Medicine (Baltimore). (2020) 99(11):e19292. 10.1097/md.0000000000019292 32176054 PMC7220113

[48] Fang T Ma C Zhang Z Sun L Zheng N. Roxadustat, a HIF-PHD inhibitor with exploitable potential on diabetes-related complications. Front Pharmacol. (2023) 14:1088288. 10.3389/fphar.2023.1088288 36843948 PMC9950780

[49] Schoene RB Swenson ER Pizzo CJ Hackett PH Roach RC Mills WJ Jr. The lung at high altitude: bronchoalveolar lavage in acute mountain sickness and pulmonary edema. J Appl Physiol (1985). (1988) 64(4):2605–13. 10.1152/jappl.1988.64.6.2605 3403445

[50] McGettrick AF O'Neill LAJ. The role of HIF in immunity and inflammation. Cell Metab. (2020) 32(4):524–36. 10.1016/j.cmet.2020.08.002 32853548

[51] Walmsley SR Print C Farahi N Peyssonnaux C Johnson RS Cramer T Hypoxia-induced neutrophil survival is mediated by HIF-1alpha-dependent NF-kappaB activity. J Exp Med. (2005) 201(1):105–15. 10.1084/jem.20040624 15630139 PMC2212759

[52] Korbecki J Simińska D Gąssowska-Dobrowolska M Listos J Gutowska I Chlubek D Chronic and cycling hypoxia: drivers of cancer chronic inflammation through HIF-1 and NF-*κ*B activation: a review of the molecular mechanisms. Int J Mol Sci. (2021) 22(19):10701. 10.3390/ijms221910701 34639040 PMC8509318

[53] Belaiba RS Bonello S Zähringer C Schmidt S Hess J Kietzmann T Hypoxia up-regulates hypoxia-inducible factor-1alpha transcription by involving phosphatidylinositol 3-kinase and nuclear factor kappaB in pulmonary artery smooth muscle cells. Mol Biol Cell. (2007) 18(12):4691–7. 10.1091/mbc.e07-04-0391 17898080 PMC2096613

[54] Saravanan S Islam VI Babu NP Pandikumar P Thirugnanasambantham K Chellappandian M Swertiamarin attenuates inflammation mediators via modulating NF-*κ*B/I *κ*B and JAK2/STAT3 transcription factors in adjuvant induced arthritis. Eur J Pharm Sci. (2014) 56:70–86. 10.1016/j.ejps.2014.02.005 24582615

[55] Cummins EP Berra E Comerford KM Ginouves A Fitzgerald KT Seeballuck F Prolyl hydroxylase-1 negatively regulates IkappaB kinase-beta, giving insight into hypoxia-induced NFkappaB activity. Proc Natl Acad Sci U S A. (2006) 103(48):18154–9. 10.1073/pnas.0602235103 17114296 PMC1643842

[56] Wang G Wang J Li X Wu Q Yao R Luo X. Hypoxia and TNF-α synergistically induce expression of IL-6 and IL-8 in human fibroblast-like synoviocytes via enhancing TAK1/NF-*κ*B/HIF-1*α* signaling. Inflammation. (2023) 46(3):912–24. 10.1007/s10753-022-01779-x 36607540

[57] El-Dessouki AM Alzokaky AA Raslan NA Ibrahim S Selim HMRM Al-Karmalawy AA. Dabigatran attenuates methotrexate-induced hepatotoxicity by regulating coagulation, endothelial dysfunction, and the NF-kB/IL-1β/MCP-1 and TLR4/NLRP3 signaling pathways. Naunyn Schmiedebergs Arch Pharmacol. (2025) 398(5):5129–45. 10.1007/s00210-024-03567-w 39527308

[58] Cao W Zeng Y Su Y Gong H He J Liu Y The involvement of oxidative stress and the TLR4/NF-*κ*B/NLRP3 pathway in acute lung injury induced by high-altitude hypoxia. Immunobiology. (2024) 229(3):152809. 10.1016/j.imbio.2024.152809 38788361

[59] Song TT Bi YH Gao YQ Huang R Hao K Xu G Systemic pro-inflammatory response facilitates the development of cerebral edema during short hypoxia. J Neuroinflammation. (2016) 13(1):63. 10.1186/s12974-016-0528-4 26968975 PMC4788817

[60] Subramaniam S Kothari H Bosmann M. Tissue factor in COVID-19-associated coagulopathy. Thromb Res. (2022) 220:35–47. 10.1016/j.thromres.2022.09.025 36265412 PMC9525243

[61] Branchford BR Carpenter SL. The role of inflammation in venous thromboembolism. Front Pediatr. (2018) 6:142. 10.3389/fped.2018.00142 29876337 PMC5974100

[62] Saha D Saha S Sergeeva EG Ionova ZI Gorbach AV. Tissue factor and atherothrombosis. Curr Pharm Des. (2015) 21(9):1152–7. 10.2174/1381612820666141013154946 25312727

[63] Wahlund CJE Çaglayan S Czarnewski P Hansen JB Snir O. Sustained and intermittent hypoxia differentially modulate primary monocyte immunothrombotic responses to IL-1β stimulation. Front Immunol. (2023) 14:1240597. 10.3389/fimmu.2023.1240597 37753073 PMC10518394

[64] Tyagi T Ahmad S Gupta N Sahu A Ahmad Y Nair V Altered expression of platelet proteins and calpain activity mediate hypoxia-induced prothrombotic phenotype. Blood. (2014) 123(8):1250–60. 10.1182/blood-2013-05-501924 24297866

[65] Ahrens I Chen YC Topcic D Bode M Haenel D Hagemeyer CE HMGB1 binds to activated platelets via the receptor for advanced glycation end products and is present in platelet rich human coronary artery thrombi. Thromb Haemost. (2015) 114(5):994–1003. 10.1160/th14-12-1073 26202300

[66] Goonewardena SN Chen Q Tate AM Grushko OG Damodaran D Blakely PK Monocyte-mediated thrombosis linked to circulating tissue factor and immune paralysis in COVID-19. Arterioscler Thromb Vasc Biol. (2024) 44(5):1124–34. 10.1161/atvbaha.122.318721 38511328 PMC11043007

[67] Neumann FJ Marx N Gawaz M Brand K Ott I Rokitta C Induction of cytokine expression in leukocytes by binding of thrombin-stimulated platelets. Circulation. (1997) 95(10):2387–94. 10.1161/01.cir.95.10.2387 9170401

[68] Mandel J Casari M Stepanyan M Martyanov A Deppermann C. Beyond hemostasis: platelet innate immune interactions and thromboinflammation. Int J Mol Sci. (2022) 23(7):3868. 10.3390/ijms23073868 35409226 PMC8998935

[69] Mertens I Verrijken A Michiels J Van der Planken M Ruige J Van Gaal L. Among inflammation and coagulation markers, PAI-1 is a true component of the metabolic syndrome. Int J Obes. (2006) 30(8):1308–14. 10.1038/sj.ijo.0803189 16389265

[70] Rijneveld AW Levi M Florquin S Speelman P Carmeliet P van Der Poll T. Urokinase receptor is necessary for adequate host defense against pneumococcal pneumonia. J Immunol. (2002) 168(7):3507–11. 10.4049/jimmunol.168.7.3507 11907112

[71] Nair V Yanamandra U Kumud R Ghosh K. PAI-1 polymorphism as a cause of severe high altitude associated arteriovenous thrombosis. Case Reports. (2016) 2016:bcr2016217361. 10.1136/bcr-2016-217361 PMC5174770 27881587

[72] Wang B Zhang YB Zhang F Lin H Wang X Wan N On the origin of Tibetans and their genetic basis in adapting high-altitude environments. PLoS One. (2011) 6(2):e17002. 10.1371/journal.pone.0017002 21386899 PMC3046130

[73] Lewis NCS Bain AR Wildfong KW Green DJ Ainslie PN. Acute hypoxaemia and vascular function in healthy humans. Exp Physiol. (2017) 102(12):1635–46. 10.1113/ep086532 28901662

[74] Pichler Hefti J Leichtle A Stutz M Hefti U Geiser T Huber AR Increased endothelial microparticles and oxidative stress at extreme altitude. Eur J Appl Physiol. (2016) 116(4):739–48. 10.1007/s00421-015-3309-3 26820158

[75] Magalhães J Ascensão A Marques F Soares J Neuparth M Ferreira R Skeletal muscle ultrastructural and plasma biochemical signs of endothelium dysfunction induced by a high-altitude expedition (pumori, 7161 m). Basic Appl Myol. (2005) 15(1):29–35.

[76] Wu A Xiong Y Li Z Liu Y Quan Q Wu L. Correlation between single nucleotide polymorphisms in hypoxia-related genes and susceptibility to acute high-altitude pulmonary edema. Genet Mol Res. (2015) 14(3):11562–72. 10.4238/2015.September.28.8 26436397

[77] Ortiz-Prado E Cordovez SP Vasconez E Viscor G Roderick P. Chronic high-altitude exposure and the epidemiology of ischaemic stroke: a systematic review. BMJ open. (2022) 12(4):e051777. 10.1136/bmjopen-2021-051777 35487749 PMC9058702

[78] Naeije R. Physiological adaptation of the cardiovascular system to high altitude. Prog Cardiovasc Dis. (2010) 52(6):456–66. 10.1016/j.pcad.2010.03.004 20417339

[79] Faeh D Gutzwiller F Bopp M. Lower mortality from coronary heart disease and stroke at higher altitudes in Switzerland. Circulation. (2009) 120(6):495–501. 10.1161/circulationaha.108.819250 19635973

[80] Basak N Norboo T Mustak MS Thangaraj K. Heterogeneity in hematological parameters of high and low altitude Tibetan populations. J Blood Med. (2021) 12:287–98. 10.2147/jbm.S294564 34040473 PMC8139737

[81] Zheng B Luo Y Li Y Gu G Jiang J Chen C Prevalence and risk factors of stroke in high-altitude areas: a systematic review and meta-analysis. BMJ open. (2023) 13(9):e071433. 10.1136/bmjopen-2022-071433 37734891 PMC10514645

[82] Jaillard AS Hommel M Mazetti P. Prevalence of stroke at high altitude (3380 m) in Cuzco, a town of Peru: a population-based study. Stroke. (1995) 26(4):562–8. 10.1161/01.STR.26.4.562 7709397

[83] Anand AC Jha SK Saha A Sharma V Adya CM. Thrombosis as a complication of extended stay at high altitude. Natl Med J India. (2001) 14(4):197–201. 11547523

[84] Jha SK Anand AC Sharma V Kumar N Adya CM. Stroke at high altitude: Indian experience. High Alt Med Biol. (2002) 3(1):21–7. 10.1089/152702902753639513 12006161

[85] De Ferrari A Miranda JJ Gilman RH Dávila-Román VG León-Velarde F Rivera-Ch M Prevalence, clinical profile, iron status, and subject-specific traits for excessive erythrocytosis in Andean adults living permanently at 3,825 meters above sea level. Chest. (2014) 146:1327–36. 10.1378/chest.14-0298 24874587 PMC4219344

[86] Champigneulle B Brugniaux JV Stauffer E Doutreleau S Furian M Perger E Expedition 5300: limits of human adaptations in the highest city in the world. J Physiol. (2024) 602(21):5449–62. 10.1113/jp284550 38146929

[87] Ma J Niu H Han C Qu Y. Quantify retinal structure in high-altitude residents with and without high altitude polycythemia. BMC Ophthalmol. (2023) 23(1):6. 10.1186/s12886-022-02674-7 36597056 PMC9811807

[88] Oberholzer L Lundby C Stauffer E Ulliel-Roche M Hancco I Pichon A Reevaluation of excessive erythrocytosis in diagnosing chronic mountain sickness in men from the world’s highest city. Blood. (2020) 136(16):1884–8. 10.1182/blood.2019004508 32614941

[89] Stauffer E Loyrion E Hancco I Waltz X Ulliel-Roche M Oberholzer L Blood viscosity and its determinants in the highest city in the world. J Physiol. (2020) 598(18):4121–30. 10.1113/jp279694 32445208

[90] Nader E Skinner S Romana M Fort R Lemonne N Guillot N Blood rheology: key parameters, impact on blood flow, role in sickle cell disease and effects of exercise. Front Physiol. (2019) 10:1329. 10.3389/fphys.2019.01329 31749708 PMC6842957

[91] Isbister JP. Hyperviscosity: clinical disorders. In: Baskurt OK Hardeman MR Rampling MW Meiselman HJ, editors. Handbook of Hemorheology and Hemodynamics. Amsterdam: IOS Press (2007). p. 371–91.

[92] Ge X Lu Y Chen S Gao Y Ma L Liu L Genetic origins and adaptive evolution of the deng people on the Tibetan plateau. Mol Biol Evol. (2023) 40(10):msad205. 10.1093/molbev/msad205 37713634 PMC10584363

[93] Bigham AW Lee FS. Human high-altitude adaptation: forward genetics meets the HIF pathway. Genes Dev. (2014) 28(20):2189–204. 10.1101/gad.250167.114 25319824 PMC4201282

[94] Witt KE Huerta-Sánchez E. Convergent evolution in human and domesticate adaptation to high-altitude environments. Philos Trans R Soc Lond B Biol Sci. (2019) 374(1777):20180235. 10.1098/rstb.2018.0235 31154977 PMC6560271

[95] Childebayeva A Harman T Weinstein J Goodrich JM Dolinoy DC Day TA DNA methylation changes are associated with an incremental ascent to high altitude. Front Genet. (2019) 10:1062. 10.3389/fgene.2019.01062 31737045 PMC6828981

[96] Wang L Liu WQ Du J Li M Wu RF Li M. Comparative DNA methylation reveals epigenetic adaptation to high altitude in snub-nosed monkeys. Zool Res. (2024) 45(5):1013–26. 10.24272/j.issn.2095-8137.2024.050 39147716 PMC11491775

[97] Parati G Revera M Giuliano A Faini A Bilo G Gregorini F Effects of acetazolamide on central blood pressure, peripheral blood pressure, and arterial distensibility at acute high altitude exposure. Eur Heart J. (2013) 34(10):759–66. 10.1093/eurheartj/ehs140 22711752

[98] Oka S-i Kamata H Kamata K Yagisawa H Hirata H. N-Acetylcysteine suppresses TNF-induced NF-*κ*B activation through inhibition of I*κ*B kinases. FEBS Lett. (2000) 472(2-3):196–202. 10.1016/S0014-5793(00)01464-2 10788610

[99] Kim H Seo JY Roh KH Lim JW Kim KH. Suppression of NF-*κ*B activation and cytokine production by N-acetylcysteine in pancreatic acinar cells. Free Radic Biol Med. (2000) 29(7):674–83. 10.1016/S0891-5849(00)00368-3 11033420

[100] Parker WA Orme RC Hanson J Stokes HM Bridge CM Shaw PA Very-low-dose twice-daily aspirin maintains platelet inhibition and improves haemostasis during dual-antiplatelet therapy for acute coronary syndrome. Platelets. (2019) 30(2):148–57. 10.1080/09537104.2019.1572880 30759035 PMC6425913

[101] Cornolo J Mollard P Brugniaux JV Robach P Richalet J-P. Autonomic control of the cardiovascular system during acclimatization to high altitude: effects of sildenafil. J Appl Physiol. (2004) 97(3):935–40. 10.1152/japplphysiol.00239.2004 15145924

[102] Poudel S Gautam S Adhikari P Zafren K. Physiological effects of sildenafil versus placebo at high altitude: a systematic review. High Alt Med Biol. (2024) 25(1):16–25. 10.1089/ham.2022.0043 37751174

[103] Kiss T Kovacs K Komocsi A Tornyos A Zalan P Sumegi B Novel mechanisms of sildenafil in pulmonary hypertension involving cytokines/chemokines, MAP kinases and akt. PLoS One. (2014) 9(8):e104890. 10.1371/journal.pone.0104890 25133539 PMC4136836

[104] Xu W Asosingh K Janocha AJ Madden E Wanner N Trotter D Hypoxia responses in arginase 2 deficient mice enhance cardiovascular health. bioRxiv 2025.02.20.639297. (2025):639297. 10.1101/2025.02.20.639297

[105] Li M Tang X Liao Z Shen C Cheng R Fang M Hypoxia and low temperature upregulate transferrin to induce hypercoagulability at high altitude. Blood. (2022) 140(19):2063–75. 10.1182/blood.2022016410 36040436 PMC10653030

[106] Shah BN Zhang X Sergueeva AI Miasnikova GY Ganz T Prchal JT Increased transferrin protects from thrombosis in Chuvash erythrocytosis. Am J Hematol. (2023) 98:1532–9. 10.1002/ajh.27021 37435906 PMC10529798

[107] Amaru R Prchal J Ganz T Zhang X Paton D Carrasco M Increased transferrin concentrations are not associated with thrombosis in people living at high altitude. J Hematol. (2025) 14:20–5. 10.14740/jh1388 39935698 PMC11809596

[108] Shah B Nocek J Zhang X Lasley P Pandit A Verda L Transferrin and protection from thrombosis in sickle cell disease. Blood. (2025) 146(Supplement 1):1357. 10.1182/blood-2025-1357

[109] Burtscher J Mallet RT Sah A Gassmann M Burtscher M Iturriaga R. Physiological differences underlying divergent hypoxia responses and altitude adaptations in humans, rats and mice. Compr Physiol. (2025) 15:e70077. 10.1002/cph4.70077 41307193 PMC12658720

[110] Song J Han S Amaru R Lanikova L Quispe T Kim D Alternatively spliced NFKB1 transcripts enriched in andean Aymara modulate inflammation, HIF and hemoglobin. Nat Commun. (2025) 16:1766. 10.1038/s41467-025-56848-0 39971917 PMC11840074

[111] Amaru R Cayo E Luna J Quispe T Prchal JT Song J. Andean Aymara enriched genetic variants are beneficial to high altitude adaptation of Andean quechuas living at 5000 m. Blood. (2022) 140:11035–6. 10.1182/blood-2022-170866

[112] Terefe E Belay G Han J Hanotte O Tijjani A. Genomic adaptation of Ethiopian indigenous cattle to high altitude. Front Genet. (2022) 13:960234. 10.3389/fgene.2022.960234 36568400 PMC9780680

[113] Scheinfeldt LB Soi S Thompson S Ranciaro A Woldemeskel D Beggs W Genetic adaptation to high altitude in the Ethiopian highlands. Genome Biol. (2012) 13:R1. 10.1186/gb-2012-13-1-r1 22264333 PMC3334582

